# Reactive Oxygen Species and Abiotic Stress in Plants

**DOI:** 10.3390/ijms21207433

**Published:** 2020-10-09

**Authors:** Tsanko Gechev, Veselin Petrov

**Affiliations:** 1 Center of Plant Systems Biology and Biotechnology, 139 Ruski Blvd., 4000 Plovdiv, Bulgaria; tsangech@uni-plovdiv.bg; 2 University of Plovdiv, 24 Tsar Assen str., 4000 Plovdiv, Bulgaria; 3 Agricultural University of Plovdiv, 12 Mendeleev str., 4000 Plovdiv, Bulgaria

**Keywords:** abiotic stress, acid toxicity, drought, heat, heavy metals, oxidative stress, salinity

## Abstract

Abiotic stresses cause plant growth inhibition, damage, and in the most severe cases, cell death, resulting in major crop yield losses worldwide. Many abiotic stresses lead also to oxidative stress. Recent genetic and genomics studies have revealed highly complex and integrated gene networks which are responsible for stress adaptation. Here we summarize the main findings of the papers published in the Special Issue “ROS and Abiotic Stress in Plants”, providing a global picture of the link between reactive oxygen species and various abiotic stresses such as acid toxicity, drought, heat, heavy metals, osmotic stress, oxidative stress, and salinity.

## 1. Introduction

Abiotic stresses, particularly drought, salinity, extreme temperatures, and pollutants, are hampering crop productivity worldwide. Furthermore, severe and/or sustained abiotic stresses can trigger the death of individual cells and even the whole plant, thus fully compromising the yield [1,2,3]. Reactive oxygen species (ROS)-mediated abiotic stress-induced programmed cell death was documented for many plant species [4]. At the same time, ROS are important signals that regulate growth, developmental processes, and stress adaptation [5,6,7].

Considerable physiological and molecular research has been done on the abiotic and oxidative stress networks [8,9,10]. As a result, it became clear that these networks are far more complex than initially suspected, and the interaction between them is intricate. Therefore, more genetic studies and systems biology approaches are needed to further elucidate the complex nature of these ROS and abiotic stress networks.

In this editorial, we summarize the main findings of the nine articles collected in the Special Issue “ROS and Abiotic Stress in Plants”. 

## 2. Genetic and Systems Biology Approaches Revealing the Complex Abiotic and Oxidative Stress Networks

High temperatures are detrimental to many crops. Wang et al. (2019) reported a pepper cultivar (17CL30) with remarkable tolerance to heat [11]. They compared the transcriptome and metabolome of 17CL30 with a heat-sensitive cultivar (05S180) and were able to identify 5754 and 5756 differentially expressed genes under heat stress in the leaves of 17CL30 and 05S180, respectively. Although the heat shock-responsive genes were induced in both cultivars, the upregulation was more pronounced in the tolerant 17CL30. The authors suggested that the higher levels of glutathione and glutathione S-transferases play a critical role in pepper response to heat shock and might contribute to the heat tolerance [11].

A critical role of glutathione was also suggested for counteracting cadmium (Cd) toxicity in *Arabidopsis thaliana* [12]. Cd and other heavy metals are major soil pollutants and can induce severe oxidative stress. Using time-course analysis of the early Cd responses (0–24 h), the authors identified rapid Cd-induced glutathione depletion in the roots, but not in leaves, which they proposed was critical for a proper “alert response”. The glutathione depletion in roots was concomitant with a quick induction of NADPH oxidases, followed by the induction of ROS-marker genes and build-up of glutathione. Moreover, 1-aminocyclopropane-1-carboxylic acid (ACC), a precursor of ethylene, accumulated first in the roots and later in the leaves. The authors proposed a spatial-temporal model of acute Cd responses, in which hydrogen peroxide/glutathione ratio serves as a signal that regulates Cd responses in roots and later in shoots [12].

The review paper by Wakeel et al. [13] is devoted to the effects of the heavy metal chromium (Cr) on plants. Cr attracts the attention because of its negative impact on agricultural lands and crop yield, which is coupled to the release of significant quantities in soils from industries and Cr mines. The article summarizes the recent findings on that topic and includes details on the mechanisms of Cr-induced oxidative stress, DNA damage and genotoxicity, ultrastructural changes and damage to the photosynthetic apparatus across various plant species. It will be of considerable interest to researchers working in the field of heavy metals but also to those investigating other forms of abiotic stress. 

In another study, focused directly on oxidative stress, Omidbakhshfard et al. (2020) investigated how the biostimulant SuperFifty (SF), prepared from the brown alga *Ascophyllum nodosum*, can fully protect against oxidative damage induced by the herbicide paraquat (PQ) in *A. thaliana* [14]. While PQ treatment alone resulted in necrotic lesions, growth inhibition, and eventual cell death in the plants, these negative effects were completely prevented by pre-treatment with SF. Oxidative stress in the PQ-treated plants induced ROS marker genes, genes involved in ROS-induced programmed cell death, and autophagy-related genes. The expression of these genes was not influenced in the PQ-treated plants that were pre-treated with SF. Furthermore, SF downregulated autophagy-related genes, such as *WRKY33*, and specifically upregulated genes related to growth, hormone signaling, carbohydrate metabolism, suggesting that the biostimulant acts, at least in part, by molecular priming. Further metabolomic analyses revealed accumulation of the stress-protective metabolite maltose and the tricarboxylic acid cycle intermediates fumarate and malate. Lipidome analysis conducted in parallel indicated that triacylglycerols (TAGs) are accumulated during oxidative stress but decline upon SF priming [14]. Overall, this study demonstrated that SF can completely prevent oxidative damage, an effect associated with transcriptome reprogramming and metabolome reconfigurations. 

As mentioned above, inhibition of growth is a common response to abiotic and oxidative stresses. Bissoli et al. [15] demonstrated that acidification by organic acids can increase the levels of ROS through activation of the NADPH oxidases Arabidopsis respiratory burst oxidase homolog D (AtRBOH-D) and Arabidopsis respiratory burst oxidase homolog F (AtRBOH-F) in *A. thaliana*, which results in growth inhibition. More importantly, they identified an activation tagged mutant *sbt4.13-1D*, which is more tolerant to acidification and in which the NADPH oxidases are not activated. The *sbt4.13-1D* mutant overexpresses the protease subtilase SBT4.13 and is defective in plasma membrane H+-ATPase [15]. In addition to acidification, the mutant is also tolerant to hydrogen peroxide and toxic cations. Although the exact mode of action of SBT4.13 is not clear, this work presents evidence for a novel pathway for growth inhibition by organic acids through ROS produced by activation of NADPH oxidases [15]. 

The traditionally examined plant organs during stress studies are leaves and roots. Much less is known about molecular processes occurring in other parts of the plant. One such example is phloem sap, whose dynamics in response to abiotic stress is relatively unexplored. Ogden et al. [16] provided one comprehensive snapshot of the protein composition of phloem sap during drought and subsequent recovery in tomato. Using a proteomic approach (LC-MS/MS), the authors detected 2558 novel or previously described proteins, involved in various biological processes. Intriguingly, 169 of these proteins changed their abundance levels during drought or recovery. The fraction of the upregulated ones includes thermotolerance and osmoprotectant production factors, participants in protein folding, carboxylic acid and amino acid metabolism as well as abscisic acid (ABA) signalling. On the other hand, some of the remarkably downregulated proteins function in processes such as cell wall modification, ceramide metabolism, mitogen-activated protein phosphorylation, etc. [16]. These results demonstrate that plant vasculature may play an active role during drought-stress adaptation. 

Systems biology methods are useful not only to provide an overview of the molecular signatures of a living system during certain conditions, but may also be utilized to dissect certain signal transduction pathways. Dong et al. [17] employed such an approach to analyse in detail the mechanisms through which *A. thaliana* shaggy-related kinases 11 and 12 (AtSK11 and AtSK12) (AT5G26751 and AT3G05840) promote root growth during mild osmotic stress [17]. The authors performed RNA-seq experiments on WT and *atsk11atsk12* mutants under three different water potential regimes and, after applying a three-step selection process, identified 2 chloroplastic and 10 nuclear mild osmotic stress-responsive genes modulated by *AtSK11* and *AtSK11*. These include mostly cell wall-related proteins such as extensins, a proline-rich protein, an extension-like family member and a xyloglucan endotransglycosylase/hydrolase. Additional promoter motif and transcription factor binding analyses revealed that most of these genes are regulated by the transcription factor LRL2 [17]. Thus, this study elucidated an important aspect of the plant adaptation mechanism to mild osmotic stress.

Due to their regulatory power, transcription factors (TFs) are often the targets of choice when studying abiotic stress tolerance and mitigation. In another work reported in this Special Issue, Li et al. [18] demonstrated that the tartary buckwheat TF basic region leucine zipper domain 5 (FtbZIP5) has the potential to improve tolerance to drought and salt in *A. thaliana*. The ectopic expression of *FtbZIP5* in Arabidopsis reduced the sensitivity to salt stress and drought, and mitigated the oxidative damage. Moreover, analysis of the expression patterns of stress-responsive genes in transgenic and control plants showed that they were significantly upregulated in the first group, thus supporting the important role of FtbZIP5. Furthermore, the protein kinase FtSnRK2.6, a member of the SnRK family, often associated with salt, drought and the stress hormone ABA, was identified as a binding partner of FtbZIP5, and the phosphorylation of FtbZIP5 by FtSnRK2 was demonstrated. This allowed the authors to propose a model of how FtbZIP5 participates in the ABA-dependent signaling pathway and may contribute to increasing abiotic stress tolerance after phosphorylation by FtSnRK2.6. 

In another article focused on a TF, Guo et al. [19] studied the functions of a WRKY protein (GarWRKY5) isolated from the salt-tolerant wild cotton species *Gossypium aridum*. They found out that silencing the homologue of *GarWRKY5* in upland cotton leads to a more pronounced salt-sensitivity when compared to wild type plants. Thereafter, they continued the functional analyses by overexpressing *GarWRKY5* in Arabidopsis and confirmed the important role of the TF in salt stress. In fact, the newly obtained mutants demonstrated a higher salt tolerance at the stages of seed germination and vegetative growth. To further delve into the molecular mechanisms involved, the transgenic Arabidopsis plants and their wild type counterparts were subjected to RNA-seq. It showed that the 253 detected differentially upregulated genes were enriched in representatives of the pathways for ROS scavenging, jasmonic and salicylic acid signaling, while ethylene response and oxidative stress-related genes were enriched among the 145 downregulated ones. The authors concluded that GarWRKY5 role in salt stress tolerance promotion is probably due to its ability to modulate components of the ROS-scavenging system, such as activating the expression of glutathione S-transferase (GST) and superoxide dismutase (SOD), as well as the jasmonic and salicylic acid-mediated signaling pathways.

## 3. Conclusions

The negative consequences on food production chains inevitably caused by global climate changes, some of which we already witness, in combination with the ongoing intensive growth of the population, will be a major risk for human well-being in the coming decades. This explains the unprecedented interest of researchers in the field of plant fitness, productivity and adaptation to adverse environmental conditions. The rationale is that the profound understanding of the responses of plants to various stressful stimuli will facilitate the development of strategies to substantially enhance crop tolerance, thus contributing to food security. Luckily, the recent advances in technologies allowing large-scale “–omics” analyses accelerate this process. The studies in this Special Issue add valuable pieces of the puzzle related to stress responses by: (i) reporting novel potent regulators and the mechanisms through which they exert their effects [15,17,18,19]; (ii) throwing light on previously unexplored components [16] or the pathways involved in particular stress types [11,12,13]; or (iii) exploring strategies to mitigate the detrimental effects [14]. We are confident that they will inspire further productive research in the future. 

## Abbreviations

ABA	Abscisic acid
ACC	1-aminocyclopropane-1-carboxylic acid
PQ	Paraquat
ROS	Reactive oxygen species
SF	SuperFifty
TF	Transcription factor
